# Caregiver Singing Intervention: do Emotions and Resistance differ between Vascular dementia and Alzheimer’s?

**DOI:** 10.1093/geroni/igab046.2455

**Published:** 2021-12-17

**Authors:** Lena Hammar, Gabriella Engström, Anna Swall

**Affiliations:** 1 Mälardalen University / Dalarna University, Västerås, Vastmanlands Lan, Sweden; 2 Dalarna University, Falun, Dalarnas Lan, Sweden

## Abstract

Persons with dementia in residential care commonly express resistance of aggressiveness. Caregivers Singing (CS) - when caregivers sing for or together with persons with dementia during caring, has shown to reduce these expressions and increase communication and cooperation. Previous studies of CS have included both persons with Alzheimer’s disease (AD) and persons with Vascular dementia (VD), but no studies have been done focusing on possible differences regarding these diagnoses. As disabilities and symptoms differ between these diagnoses, the emotions and expressions, such as resistance may differ regarding response to CS. This polit study aims to describe emotions and resistiveness to care among persons with vascular or Alzheimer’s disease. Participants were five persons with AD and five persons with VD living at two different nursing homes. Video observations (VIO) occurred with them and their caregivers during morning care situations four times without CS and four times with CS. In all, 80 VIOs were rated using the Observed Emotion Rating Scale and the Resistiveness to Care Scale. These were then analyzed with descriptive statistics. Results revealed that for both persons with AD and VD, the positive emotion pleasure were never observed without CS while with CS it increased for both groups. In contrast to the positive emotion effect of CS, the negative emotions and resistiveness decreased more for persons with VD than for persons with AD. For persons with VD, the number of observations without anger increased, while observation without anger or anxiety/fear for persons with AD remind the same.

